# Welcome to *Cell Regeneration*

**DOI:** 10.1186/s13619-020-00050-8

**Published:** 2020-06-02

**Authors:** Ye-Guang Chen, Ying Lou

**Affiliations:** 1 Tsinghua University, Beijing, China; 2 The Chinese Society for Cell Biology, Shanghai, China

*Cell Regeneration*, an open-access academic journal, was relaunched by the Chinese Society for Cell Biology in 2019 (Chen and Lou 2019). This journal, founded in 2012, has published many outstanding papers in the past few years.

As an official journal of the Society, *Cell Regeneration* has undergone major actions such as reorganization of the editorial board and reorientation of journals with new scopes. In 2020, the Society and Springer began co-publishing *Cell Regeneration*, aiming to serve the authors and the readers better.

To further provide efficient, effective, and equitable peer-review and publishing services, we are continually working on improving the efficiency of the core links in editorial workflow. This year we will try the following measures:

1. On the premise that submission to *Cell Regeneration* conforms to all editorial policies recognized by COPE, WAME, and ICMJE, the submission process to *Cell Regeneration* will be simplified. In particular, we will not have a strict requirement on the format of the first submission. If a manuscript can clearly show the scientific rigour, our editors and reviewers will focus on the importance and innovation of the work described in it. By doing so, we hope to reduce the authors’ workload and accelerate the review process. A stringent format requirement will be applied only to the manuscripts ready to be accepted.
2. We try to limit the peer review process to one round. Our handling editors make a judgment on whether a manuscript should be sent out for peer review with evident editorial standards when it is submitted to *Cell Regeneration*. Reviewers are expected to provide objective and constructive comments to help the authors to improve their overall work. After the revision is returned, the handling editor may recommend a final decision with their careful evaluations and decisive judgments at the end of the first round of revision based on the reviewers’ reports and the authors’ responses, and try to avoid multiple rounds of peer review.

In the past year, we have received many excellent articles. For example, in the thematic series “Gene editing and stem cells”, Xingxu Huang and Guang Yang reviewed the advances of genome editing technologies and the derivative technologies using the CRISPR/Cas system as well as their applications in the studies of stem cells and regeneration (Yang and Huang 2019). In their review paper, Ling Shuai and colleagues summarized possible developmental models of the first cell fate decision and discussed the signaling pathways and transcriptional networks regulating this process (Yao et al. 2019). Feng Gu's lab published an original research article describing a PAM identification system termed PAM-DOSE (PAM Definition by Observable Sequence Excision), and this method can facilitate the identification of functional PAMs for Cas-mediated human genome editing applications (Tang et al. 2019). This series is still open for submissions of original research articles and reviews.

In addition, the articles on other topics have also been accepted. For instance, Abo-Aziza and colleagues explored a possible application of stem cells in treatment of hydatidosis, a worldwide zoonotic disease caused by Echinococcus that creates damages in multiple organs including the liver. The authors reported that bone marrow mesenchymal stem cell transplantation following albendazole administration can regenerate injured liver tissue in experimentally infected rats (Abo-Aziza et al. 2019).

Before this series started, we had published Xiaofen Zhong and Yiping Zhong's work on the design of a retrograde monosynaptic tracing with CRISPR/Cas9-mediated targeting to efficiently analyze transplant connectivity for the comprehensive assessment of host-donor cell innervation (Xing et al. 2019). Wenjuan Li and coworker reported an interesting finding that the nuclear-cytoplasmic shuttling of class IIa histone deacetylases is important for somatic cell reprogramming as trapping them in the nucleus enhances the early phase of reprogramming but is deleterious afterwards (Luo et al. 2019). Huntington's disease (HD) is an autosomal dominant neurodegenerative disorder that is caused by the polyglutamine repeat in the N-terminal region of the huntingtin due to the CAG repeat expansion in the exon 1 of its gene. In a review paper, Xiao-Jiang Li and colleagues discussed the application of large animal models in neurodegenerative disease research, especially with the HD large animal models as the large animal models are likely to mimic important neuropathological features in humans (Yan et al. 2019). The heart in adult humans loses the regeneration ability as cardiomyocytes cannot proliferate, and thus ways to reactivate cardiomyocyte division have a great potential to treat heart failure. Satwat Hashmi and H.R. Ahmad summarized the molecules and signaling pathways related to the cell cycle control of cardiomyocytes and the possible mechanisms to trigger myocardial regeneration in their review paper (Hashmi and Ahmad 2019).

Starting this year, Cell Regeneration is published by Spring Open via collaboration with Springer Nature. Through the great platform, we will strive to our high standards to publish the work at the forefront of basic and applied research of stem cell biology, regenerative biology and regenerative medicine, including embryonic stem cells, induced pluripotent stem cells, tissue stem cells, tissue and organ regeneration, related methodology, biomaterials, regenerative medical applications and associated research. Indeed, several high-quality papers are in the production pipeline. Jingwen Wang, Junjiu Huang, and Guang Shi summarized expression patterns, functions and regulation of retrotransposons in PSCs and early embryonic development. Yuan Liu, Xiaochen Xiong, and Ye-Guang Chen highlighted a recent work by Murata et al on intestinal epithelium plasticity through Ascl2-dependent dedifferentiation of absorptive and secretory precursors in mice. Pooja Kulkarni's study on autologous bone marrow mononuclear cell transplantation in patients with chronic traumatic brain injury demonstrated the safety and efficacy of cell transplantation in chronic TBI. Itali Linero Segrera's systematic review and meta-analysis showed that the application of mesenchymal stem cells-conditioned medium to bone defects has a positive and favorable effect on the repair and regeneration of bone tissue, suggesting the possible application in clinical practice in near future.

In summary, we endeavor to accelerate our review process and publish high quality papers on stem cells and regeneration medicine and would like to invite you to submit your manuscripts to Cell Regeneration.
